# Astrocyte store-operated calcium entry is required for centrally mediated neuropathic pain

**DOI:** 10.1097/PR9.0000000000001484

**Published:** 2026-07-17

**Authors:** Mariya A. Prokhorenko, Jeremy T. Smyth

**Affiliations:** a Neuroscience Graduate Program, F. Edward Hébert School of Medicine, Uniformed Services University of the Health Sciences, Bethesda, MD, USA; b Henry M. Jackson Foundation for the Advancement of Military Medicine, Bethesda, MD, USA; c Department of Anatomy, Physiology, and Genetics, F. Edward Hébert School of Medicine, Uniformed Services University of the Health Sciences, Bethesda, MD, USA

**Keywords:** Chronic pain, Calcium signaling, Astrocyte, Drosophila, Stim, Orai

## Abstract

**Introduction:**

Acute injuries transition to chronic neuropathic pain through central sensitization (CS), which involves plastic changes to nociceptive networks within the central nervous system. Astrocytes are essential mediators of CS, and many neuromodulatory functions of astrocytes are regulated by complex Ca^2+^ signaling mechanisms. The role of astrocyte Ca^2+^ signaling in CS and the specific astrocyte Ca^2+^ channels involved are poorly defined.

**Objectives:**

Our objective was to test the role of store-operated Ca^2+^ entry (SOCE), a highly conserved Ca^2+^ signaling mechanism that mediates Ca^2+^ influx in response to depletion of endoplasmic reticulum Ca^2+^ stores, in CS and neuropathic pain.

**Methods:**

We used a *Drosophila* model of CS based on the development of thermal allodynia 7 days after acute nerve injury, combined with a transcriptional reporter of intracellular Ca^2+^ mobilization. In vivo, astrocyte-specific RNAi was then used to test the role of Orai SOCE channels and Orai-activating Stim proteins in injury-induced astrocyte Ca^2+^ signaling and thermal allodynia.

**Results:**

Astrocytes exhibited Stim and Orai-dependent Ca^2+^ signaling 3 to 4 days postinjury. Astrocyte-specific suppression of Stim and Orai completely inhibited the development of thermal allodynia 7 days after injury and also inhibited the loss of GABAergic inhibitory neurons required for CS in flies as well as mammals. Finally, constitutive SOCE-mediated astrocyte Ca^2+^ signaling resulted in thermal allodynia even in the absence of injury.

**Conclusion:**

Astrocyte SOCE is necessary and sufficient for CS and thermal allodynia in *Drosophila*, adding key new understanding to the conserved astrocyte Ca^2+^ signaling mechanisms involved in development of neuropathic pain.

## Abstract 2

Supplemental Digital Content is Available in the Text.

Astrocyte Ca^2+^ signaling mediated by store-operated Ca^2+^ entry is required for chronic pain and may be an important target for nonopioid pain therapeutics.

## 1. Introduction

Acute nerve injuries can transition into chronic neuropathic pain through the process of central sensitization (CS). Central sensitization involves changes to neural connectivity, synaptic plasticity, and response thresholds that sensitize nociceptive circuits within the central nervous system (CNS).^1,8^ In addition to neurons, astrocytes are also essential mediators of CS and through their ability to both respond to and modulate neural function.^22,27,35^ Intracellular Ca^2+^ signaling in astrocytes is essential for this bi-directional communication with neurons. Astrocyte Ca^2+^ signals are stimulated by synaptic neurotransmitters, and these Ca^2+^ signals regulate many of the neuromodulatory functions of astrocytes.^37^ A canonical mechanism for initiation of astrocyte Ca^2+^ signaling involves stimulation of G_q_-coupled metabotropic neurotransmitter receptors, which results in Ca^2+^ release from endoplasmic reticulum stores via inositol 1,4,5-trisphosphate receptors (IP_3_R),^42^ and both the type-5 metabotropic glutamate receptor (mGluR5) and type-2 IP_3_ (IP_3_R2) are required specifically in astrocytes for CS. Importantly, however, Ca^2+^ influx channels are also essential for the neuromodulatory function of astrocytes but the role of astrocyte Ca^2+^ influx channels specifically in chronic pain has not been addressed.

In this study, we tested the hypothesis that Orai Ca^2+^ influx channels are required in astrocytes for CS and neuropathic pain. Orai channels are activated by depletion of endoplasmic reticulum (ER) Ca^2+^ stores through the process of store-operated Ca^2+^ entry (SOCE).^38^ Endoplasmic reticulum Ca^2+^ store depletion is detected by Stim proteins that function as ER Ca^2+^ sensors, and upon store depletion, Stim activates Orai channels in the plasma membrane to mediate SOCE.^33^ Store-operated Ca^2+^ entry channels are canonically activated downstream of IP_3_R-mediated ER Ca^2+^ release. Importantly, however, Ca^2+^ signals mediated by SOCE channels can regulate signaling processes that are distinct from those mediated by IP_3_Rs.^10,16^ Store-operated Ca^2+^ entry channels can also be targeted therapeutically^6,7^ and may represent important targets for chronic pain intervention. We addressed the involvement of astrocyte SOCE-mediated Ca^2+^ signaling using a novel *Drosophila* model of CS, and our results demonstrate that astrocyte SOCE, which is highly conserved between *Drosophila* and mammals, is an essential signaling axis that underlies chronic pain.

## 2. Materials and methods

### 2.1. Materials

A table listing all materials and reagents with source information used in this study is included in Supplementary Materials (Supplemental Table 1, http://links.lww.com/PR9/A429).

### 2.2. Fly genetics & husbandry

*Drosophila* were housed in vials on standard cornmeal-agar fly food. Experimental groups were separated into vials by sex in groups of 10 to 12. Flies were 5 to 8 days old at the time of leg amputation injury. All flies kept at 25°C with a 12-hour light/dark cycle. Leg amputations, behavioral experiments, and dissections were conducted at the same morning timepoints.

### 2.3. Thermal allodynia assay

Thermal nociception assays were conducted by placing groups of 10 to 12 flies in a round chamber 3 mm high and 35 mm diameter (Vellum translucent paper glued to a 3D printed ring) on a heat block (Benchmark Scientific; Sayreville, NJ) set to 38°, 24°, or 42°C and after 60-second acclimation, fly behavior was recorded for 90 seconds at 30 frames/sec on a Google Pixel smartphone. The heat block was calibrated using an infrared thermometer (Fisher Scientific; Waltham, MA). Videos were cropped and converted to AVI files using MATLAB. Experimental genotypes were divided into 4 replicate groups (2 male, 2 female) of 10 to 12 flies to be injured or to be sham. 38°C experiments were conducted on days −1, 1, and 7 relative to injury. On day 0, flies designated as injured had the right middle leg amputated mid-femur under CO_2_ anesthesia. Sham flies were placed under CO_2_ anesthesia alone. Blinded videos were manually analyzed using FIJI and jump number and the start and stop frames of jumps were recorded. Due to right skewedness from large numbers of flies with zero jumps, data were analyzed using nonparametric Kruskal–Wallis tests with Dunn correction for multiple comparisons. Separate analysis of males and females indicated no sex differences, and male and female data were, therefore, pooled. For linear discriminant analysis (LDA), each fly's behavior was coded into a time series based on jump and roll start and stop frames using R. Time series were transformed into frequency domain spectra via fast Fourier transform and then into time domain cepstra via inverse Fourier transform. Using CepLDA in R,^20^ cepstral Fisher LDA was run by first training the model to find the best weight vectors to separate sham and injured control, then running the LDA model on injured test groups.

### 2.4. Dissection and immunohistochemistry

Dissection and immunohistochemistry of adult *Drosophila* CNS were performed as described.^15^ Briefly, cold-anesthetized flies were transferred to a 9-well glass plate on ice, rinsed in 70% ethanol, twice rinsed in S2 media (Fisher Scientific), and dissected in cold S2 on silicone (Sylgard 184, Sigma; St. Louis, MO). Dissected ventral nerve cords (VNCs) were transferred into 2% paraformaldehyde in S2 and nutated at room temperature (RT) for 1 hour and then washed 4 times for 10 minutes in phosphate buffered saline (PBS) with 0.5% Triton X-100 (PBT) with nutating at RT. Ventral nerve cords were then incubated in blocking buffer (5% bovine serum albumin in PBT) for 1 hour with nutating at RT, transferred to primary antibody in blocking buffer, and nutated at RT for 4 hours then at 4°C for 48 hours. Ventral nerve cords were washed 4 times for 15 minutes in PBT, transferred into secondary antibody at RT for 4 hours then at 4°C for 72 hours, and washed 4 times. Before mounting, a cover-glass was dipped overnight in PLL solution (poly-l-lysine hydrobromide powder, 32 mL of diH_2_O, 64 µL Kodak Photo-Flo) and stored in dark at 4°C. After secondary antibody, VNCs were transferred into 4% paraformaldehyde in PBS with nutating for 4 hours at RT, washed 4 times in PBT for 15 minutes, and then once in PBS for 10 minutes. Up to 20 VNCs were mounted onto PLL-dipped cover-glass in PBS, the cover-glass with mounted VNCs was dipped in diH_2_O, then dehydrated in successive 10 minutes baths of ethanol: 30%, 50%, 75%, 95%, 100%, 100%, 100%. DPX (Electron Microscopy Sciences; Hatfield, PA) was added to the cover glass until all samples were covered, then the cover glass was flipped onto a glass slide with 2 cover glass spacers <20 mm apart, and the slide cured at RT for 3 days before imaging.

### 2.5. GABA imaging and analysis

Samples were imaged with a Nikon A1R confocal microscope using a 40X, 1.3 NA oil objective. Z-stacks at 0.4-µm intervals were collected. Blinded image files were processed and analyzed using Fiji, and Z-stacks were converted to maximum intensity projections over the depth of the VNC. Because GABA immunofluorescence appeared as punctate structures, we determined the total area of GABA punctae by adjusting image threshold to include maximum fluorescent particles and minimum noise, converting to a binary mask, and removing small noise particles. Particles were analyzed with the following settings: 0-infinity µm^2^, 0 to 1.0 circularity, summarize, and the total fluorescence area (µm^2^) was reported. Data were compared using a nonparametric mixed-effects analysis with Šídák correction for multiple comparisons because data did not meet normality or homoscedasticity requirements.

### 2.6. Transcriptional reporter of intracellular Ca^2+^ imaging and analysis

Samples were imaged using a 60X, 1.4 NA oil objective and Z-stacks at 0.5-µm intervals were collected. Maximum intensity projections images were processed in Fiji using Calculator Plus, and the 488-nm channel for Transcriptional Reporter of Intracellular Ca^2+^ (TRIC) was divided by the 568-nm channel for total astrocyte fluorescence and then multiplied by 10 for easier visualization. A circular region of interest covering 58,436 pixels^2^ was used to measure mean intensity at 5 locations per image, covering each of the 4 bulbs and the center of the VNC, and the average of the 5 region of interest means is reported. Because the data were normally distributed but showed unequal variances, we used Welch ANOVA with Dunnett T3 correction for multiple comparisons.

## 3. Results

### 3.1. Leg amputation injury leads to thermal allodynia in adult flies

**Figure 1.**
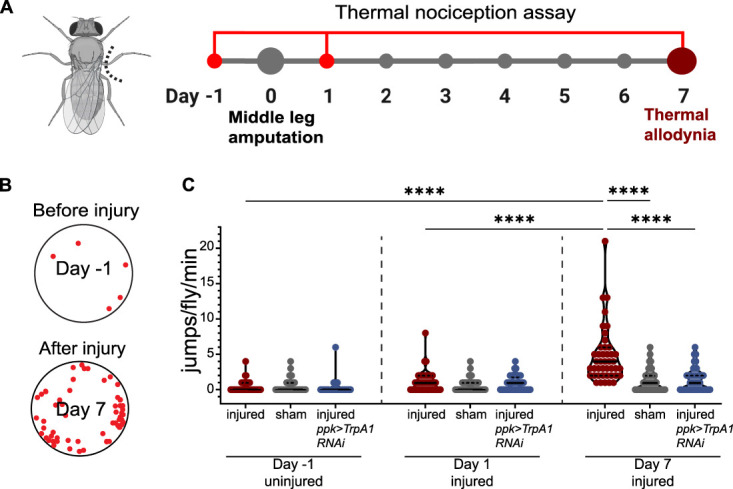
Leg amputation drives thermal allodynia in adult *Drosophila*. (A) Left: Schematic illustration of nerve injury via middle leg amputation of adult *Drosophila*. Right: Timeline of behavioral experiments to assess thermal allodynia. As indicated by the red symbols, the same cohorts of animals were tested one day before injury (day −1), and 1 and 7 days after injury. (B) Summary diagram of 1 minute of jumps, represented as red dots, of a group of 11 cohoused flies before injury and 7 days after middle leg amputation in an overhead view of a circular chamber on a 38°C hotplate. (C) Plot of the number of jumps per fly per minute at 38°C for 3 cohorts of animals on the indicated days respective to injury. The 3 cohorts are injured controls (w^1118^; alrm-GAL4), sham controls (w^1118^; alrm-GAL4), and injured with sensory neuron–specific TrpA1 suppression with ppk-GAL4 driven TrpA1 RNAi. Each symbol represents the total number of jumps for a single animal (n ≥ 43). *0.01 < *P* < 0.05; ****P < 0.0001, Kruskal–Wallis with Dunn multiple comparisons.

We adapted methods for modeling CS in adult *Drosophila* from Khuong et al.^17^ to analysis of astrocyte Ca^2+^ signaling mechanisms. These methods are based on the development of thermal allodynia, a form of hypersensitivity and neuropathic pain in which a normally nonpainful temperature is perceived as painful. Adult flies exhibit escape behaviors in the form of jumps placed on surface temperatures that they perceive as noxious or painful. As shown in Figure 1A-C and Supplemental Figure S1, http://links.lww.com/PR9/A429, healthy uninjured flies exhibit relatively few jumps on surface temperatures from 24°C to 38°C, whereas repeated jumping behavior is exhibited by flies on a 42°C surface. This indicates that in healthy flies, 42°C is perceived as painful, whereas 38°C is below the pain threshold. Jumping at 42°C was completely inhibited in animals with *ppk-GAL4*-driven, sensory neuron-specific suppression of the thermal-sensitive TrpA1 ion channel (Supplemental Figure S1, http://links.lww.com/PR9/A429), verifying that jumping is a thermal nociceptive response. We then introduced nerve injury by middle leg amputation as illustrated in Figure 1A and quantified jumping behavior at subthreshold 38°C to assess the development of thermal allodynia. As shown in Figure 1C, injured animals did not exhibit increased jumping 1 day after injury compared with uninjured controls, indicating that injury did not result in acute hypersensitivity. Seven days after injury, however, flies now exhibited significantly more jumps at normally subnoxious 38°C compared with uninjured sham controls (Fig. 1A-C). This hypersensitivity to 38°C is indicative of thermal allodynia and suggests development of CS over the 7 days postinjury. Jumping of injured animals at 38°C was again prevented by sensory neuron–specific TrpA1 suppression (Fig. 1C), demonstrating a thermal nociceptive response. We did not conduct thermal sensitivity assays longer than 7 days postinjury; however, Khuong et al.^17^ demonstrated that thermal allodynia in this model is chronic and long-lasting, persisting for at least 21 days.

### 3.2. Store-operated Ca^2+^ entry-dependent astrocyte Ca^2+^ signaling is activated after injury

**Figure 2.**
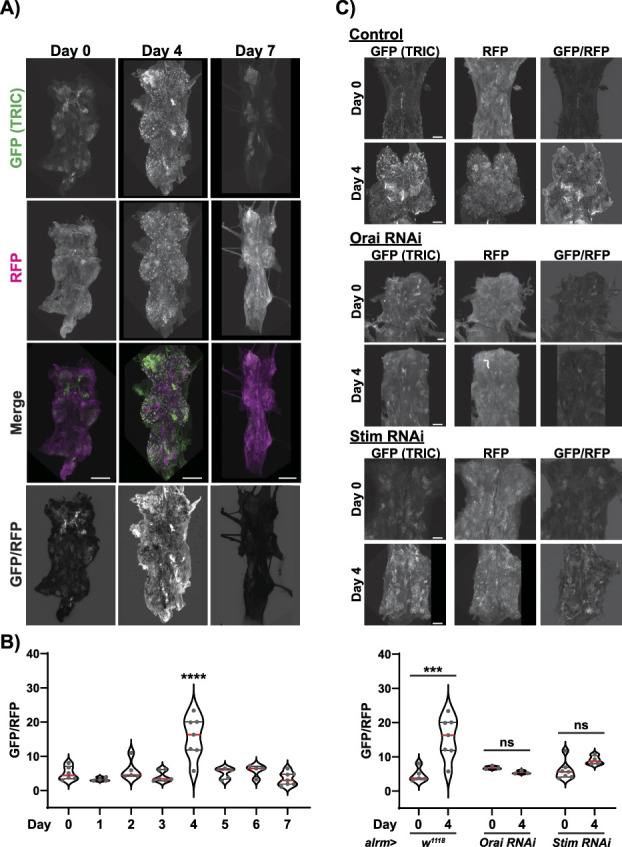
Leg amputation injury activates SOCE-dependent Ca^2+^ signaling in VNC astrocytes. (A) Representative images of TRIC-mediated GFP fluorescence (green) and astrocyte-restricted RFP fluorescence (magenta) in VNCs from uninjured (day 0) and day 4 and day 7 postleg amputation injury animals. Also shown are images of the product of GFP divided by RFP fluorescence (GFP/RFP), representative of the normalized Ca^2+^ signaling activity reported by TRIC. Scale bars = 50 µm. (B) Plot of the mean GFP/RFP fluorescence ratio from VNCs from animals on each day after injury up to day 7. Each symbol represents a single VNC (n ≥ 4 for each day). *P < 0.05, Welch ANOVA and multiple comparisons with Dunnett T3 correction. (C) Representative images of TRIC-mediated GFP fluorescence (green), astrocyte-restricted RFP fluorescence (magenta), and GFP/RFP ratio from VNCs cropped to show the anterior 2 bulbs from uninjured (day 0) and day 4 injured control (w^1118^; alrm-GAL4), alrm-GAL4 > Stim RNAi, and alrm-GAL4 > Orai RNAi animals. Scale bars = 25 µm. (D) Plot of the mean GFP/RFP fluorescence ratio from day 0 and day 4 injured control, Stim, and Orai RNAi VNCs. Each symbol represents a single VNC. *P < 0.05, Welch ANOVA and multiple comparisons with Dunnett T3 correction; ns, not significant. Red lines in violin plots represent medians, and gray lines represent quartiles. GFP, green fluorescent protein; RFP, red flourescent protein; SOCE, store-operated Ca^2+^ entry; TRIC, transcriptional reporter of intracellular Ca^2+^; VNC, ventral nerve cord.

We next determined whether leg amputation injury in flies results in astrocyte Ca^2+^ signaling within the VNC, the *Drosophila* analog of the mammalian spinal cord. Because we could not predict a priori when astrocyte Ca^2+^ signals may occur postinjury, we used the Transcriptional reporter of intracellular Ca^2+ ^(TRIC) indicator expressed in astrocytes with the previously validated, astrocyte-specific *alrm-GAL4* driver.^11^ TRIC couples green fluorescent protein (GFP) expression to intracellular Ca^2+^ mobilization through expression of a split transcriptional activator that is reconstituted by Ca^2+^ binding. TRIC functions as a Ca^2+^ “memory” indicator that provides a quantitative readout of Ca^2+^ signals over 12 to 24 hours.^12^ We imaged GFP fluorescence in VNCs of animals that expressed TRIC transgenes specifically in astrocytes on each of the 7 days after injury. Astrocytes also expressed membrane-bound red flourescent protein (RFP) to label astrocytes independently of Ca^2+^ activity, allowing us to calculate a ratio of GFP to RFP fluorescence and factor out Ca^2+^-independent changes. Astrocyte GFP fluorescence and the GFP/RFP ratio remained stably low from day zero (uninjured) until day 4 postinjury, when a highly reproducible spike in astrocyte GFP fluorescence and GFP/RFP ratio was seen throughout the VNC (Fig. 2A, B). This spike was confined to day 4 and returned to near-uninjured levels from days 5 to 7. These results suggest that leg amputation injury results in Ca^2+^ mobilization in VNC astrocytes during the 3- to 4-day timeframe postinjury. We next determined whether injury-induced astrocyte Ca^2+^ mobilization on day 4 postinjury requires SOCE by suppressing Stim and Orai expression using validated RNAi constructs previously demonstrated to specifically reduce *Stim* and *Orai* transcript levels by 72% and 80%, respectively.^32^ *Alarm-GAL4*-driven *Stim* and *Orai* RNAi did not significantly affect TRIC-mediated GFP expression in astrocytes or the GFP/RFP ratio in the absence of injury (Fig. 2C,D, Day 0), but remarkably the increase in GFP expression and GFP/RFP ratio on day 4 postinjury was strongly suppressed in animals with astrocyte-specific *Stim* and *Orai* RNAi (Fig. 2C,D). These results suggest that astrocyte Ca^2+^ signaling triggered by nerve injury requires SOCE.

### 3.3. Astrocyte store-operated Ca2+ entry is required for injury-induced thermal allodynia

**Figure 3.**
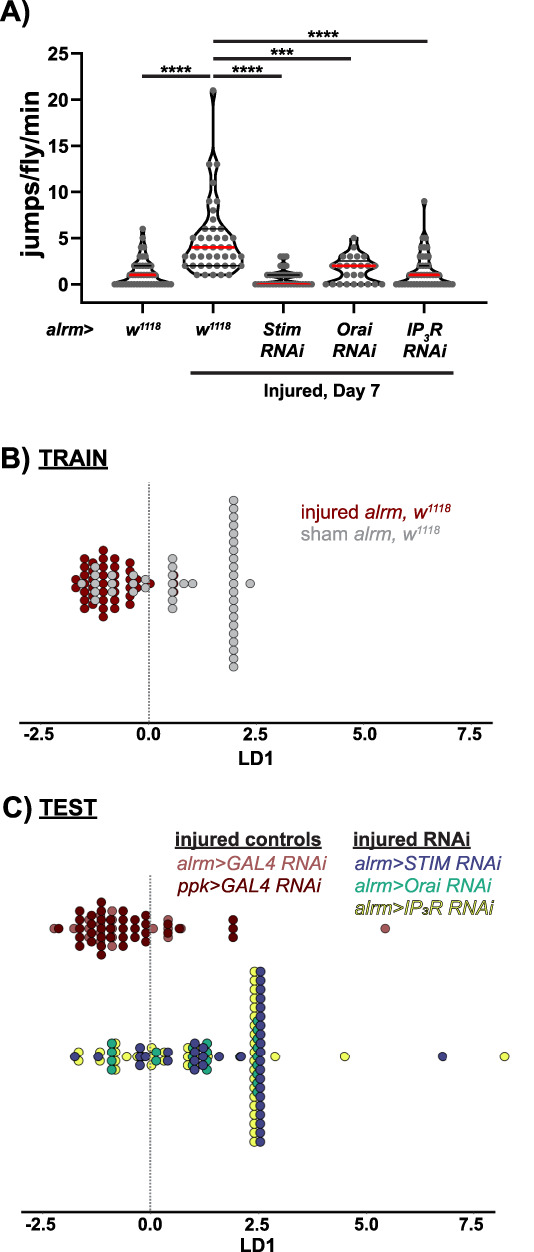
Astrocyte SOCE is necessary for injury-induced thermal allodynia. (A) Plot of the number of jumps per fly per minute at 38°C on day 7 after leg amputation injury (“injured”) for the indicated genotypes. Also shown are data for uninjured controls (left-most dataset). Each symbol represents the total number of jumps for a single animal (N = 45 for uninjured alrm > w^1118^ controls, 43 for injured alrm > w^1118^ controls, 38 for Stim RNAi, 25 for Orai RNAi, and 45 for IP_3_R RNAi). ****P < 0.0001, ***P = 0.0001, Kruskal–Wallis with Dunn multiple comparisons. (B and C) Cepstral linear discriminant analysis based on graded behavior over 1 minute at 38°C for uninjured and 7-day injured animals with indicated genotypes. (B) The result of training the model using data from sham and injured alrm > w^1118^ animals. The dotted line presents the linear decision boundary, and each symbol represents the linear discriminant or “decision” of the model for each animal based on the deconvolved time series for that animal (classification rate = 0.80). Note that the sham animals cluster to the right of the decision boundary whereas the injured animals cluster to the left, defining right as uninjured behavior and left as injured behavior. (C) Results using test data from 2 groups of injured controls expressing nontargeting RNAi (alrm > GAL4 RNAi and ppk > GAL4 RNAi) and injured animals with astrocyte expression of Stim, Orai, and IP_3_R RNAi. Note that the Stim, Orai, and IP_3_R RNAi animals cluster to the right of the decision boundary consistent with uninjured behavior, despite these animals being injured. SOCE, store-operated Ca^2+^ entry.

Suppression of injury-induced astrocyte Ca^2+^ responses by Stim and Orai knockdown suggests that astrocyte SOCE may also be required for the development of centrally mediated thermal allodynia. We tested this with thermal allodynia assays in animals expressing *Stim* and *Orai* RNAi specifically in astrocytes. We also suppressed IP_3_R as a positive control for an astrocyte Ca^2+^ channel known to be required for development of CS.^18^ Before injury, jumps at 24°C and 38°C in astrocyte *Stim*, *Orai*, and *IP*_*3*_*R* RNAi groups were relatively few and no different from control animals (Supplemental Figure S2A, B, http://links.lww.com/PR9/A429), indicating that suppression of these targets alone does not alter jumping behavior. Seven days after injury, control animals exhibited significantly more jumps at 38°C compared with uninjured controls as expected (Fig. 3A). In striking contrast, however, jumping at 38°C 7 days after injury in animals with astrocyte-specific *Stim* and *Orai* RNAi, as well as *IP*_*3*_*R* RNAi, was nearly completely suppressed and was no different from uninjured controls (Fig. 3A). This suggests that as for IP_3_R, astrocyte Ca^2+^ signaling mediated by Stim and Orai is required for nerve injury–induced thermal allodynia. Suppression of jumping was not due to an overall block of thermal nociception because uninjured *Stim*, *Orai*, and *IP*_*3*_*R* RNAi animals exhibited frequent jumps at 42°C (Supplemental Figure S2C, http://links.lww.com/PR9/A429).

We found in thermal allodynia assays that injured fly behavior at 38°C is more complex over the 1-minute time series than a simple count of jump number can reflect. For example, some jumps are frequent and clustered in a short period of time, or are spread out over the entire minute, and some flies exhibit responses more severe than jumping such as rolling or seizure-like activity. To account for this complexity, we reanalyzed the videos used for jump counts of 7-day injured flies in Figure 3A by binning the behavior of each fly, per frame of video, into 3 graded categories to form time series. In this grading scheme, one was assigned to all nonescape or nonavoidance activities (eg, walking, grooming), 2 was assigned to jumping, and 3 was assigned to rolling, seizure-like responses (Supplemental Figure S3A, http://links.lww.com/PR9/A429). Using cepstral Fisher discriminant analysis,^20^ we then trained a discriminant model using time series data from injured and sham *w*^*1118*^*; alrm-GAL4* controls animals. In Figure 3B, each fly's estimated discriminant is plotted along the first linear discriminant (LD1) axis, with the linear decision boundary marked as a dotted line at a LD1 value of 0. This resulted in a majority of sham animals' discriminants having positive values relative to the decision boundary and a majority of injured animals having negative values, with a highly accurate classification rate of 0.80. The model was then tested using 2 injured fly groups expressing control RNAi and 3 injured fly groups expressing *Stim*, *Orai*, or *IP*_*3*_*R* RNAi in astrocytes. The model classified most flies from the 2 injured control groups with negative discriminants as expected. In contrast, the model assigned positive discriminants to 81.58% of *Stim* RNAi, 70.83% of *Orai* RNAi, and 68.89% of *IP*_*3*_*R* RNAi injured flies, consistent with sham animal behavior despite these animals being injured (Fig. 3C). Thus, even when considering behavior patterns more complex than jumping, astrocyte-specific *Stim*, *Orai*, and *IP*_*3*_*R* suppression prevents behaviors associated with thermal allodynia after injury.

### 3.4. Astrocyte store-operated Ca2+ entry channels are required for injury-induced loss of inhibitory GABA neurons

**Figure 4.**
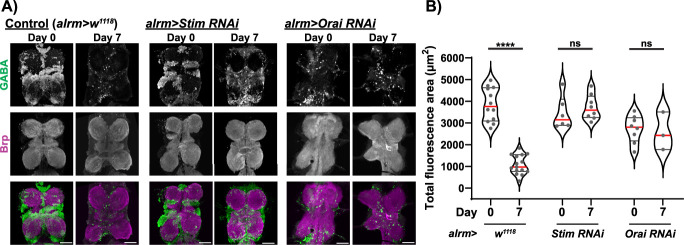
Astrocyte SOCE is required for injury-induced loss of GABAergic neurons. (A) Representative images of VNCs from uninjured (day 0) or day 7 injured control (alrm; w^1118^; left), alrm > Stim RNAi, and alrm > Orai RNAi animals labeled with antibodies to GABA (green) and Bruchpilot (magenta), which labels synaptic active zones within the neuropil and reveals the overall morphology of the VNCs. Scale bars = 50 µm. (B) Plot of the total anti-GABA fluorescence area from VNCs of uninjured (day 0) or day 7 injured animals with the indicated genotypes. Each symbol represents a single VNC. ****P < 0.0001; ns, not significant; mixed effects analysis, multiple comparisons with Šídák correction. Red lines in violin plots represent medians, and black lines represent quartiles. SOCE, store-operated Ca^2+^ entry.

Sensory neurons of fly middle legs project onto the second VNC bulbs, and it was shown that inhibitory GABAergic neuron loss in the VNC is necessary and sufficient for thermal allodynia in response to leg amputation.^17^ This is consistent with mammalian neuropathic pain models where reduction of tonic GABAergic inhibition is a necessary component of the neurocircuitry changes involved in CS.^21^ We also observed significant reduction in GABA-positive cell bodies within VNCs of 7-day postinjury animals compared with uninjured controls (Fig. 4A), further confirming that neuropathic pain in our model is mediated by conserved, centrally mediated neural adaptations. Strikingly, reduction of GABA-positive cells was suppressed by astrocyte-specific *Stim* and *Orai* RNAi (Fig. 4A,B). We did find that GABA positivity was modestly reduced in VNCs from uninjured *Orai* RNAi animals compared with uninjured controls, but there was no further reduction in GABA with injury in *Orai* RNAi samples.

### 3.5. Astrocyte store-operated Ca2+ entry is sufficient for thermal allodynia

**Figure 5.**
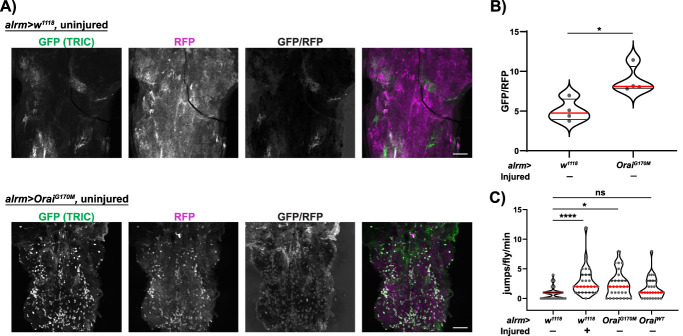
(A) Representative images of TRIC-mediated GFP fluorescence (green) and astrocyte-restricted RFP fluorescence (magenta) in VNCs from uninjured control (alrm; w^1118^) and uninjured alrm > dOrai^G170M^ animals. Also shown are images of the product of GFP divided by RFP fluorescence, representative of the normalized Ca^2+^ signaling activity reported by TRIC. Scale bars = 25 µm. (B) Plot of the mean GFP/RFP fluorescence ratio from uninjured control (alrm; w^1118^) and alrm-dOrai^G170M^ VNCs. Each symbol represents a single VNC. *P < 0.05, 2-tailed Mann–Whitney *U* test. Red lines in violin plots represent medians, and black lines represent quartiles. (C) Plot of the number of jumps per fly per minute at 38°C for uninjured animals with the indicated genotypes, as well as day 7 injured controls. Each symbol represents the total number of jumps for a single animal (n = 30 for alrm; w^1118^, 30 for dOrai^G170M^, 29 for dOrai^+^, and 27 for injured alrm; w^1118^), *0.01 < *P* < 0.05; ***P < 0.0001; ns, not significant; Kruskal–Wallis with Dunn multiple comparisons. GFP, green fluorescent protein; RFP, red flourescent protein; SOCE, store-operated Ca^2+^ entry; VNC, ventral nerve cord.

Finally, we asked whether astrocyte Ca^2+^ signaling mediated by SOCE is sufficient for thermal allodynia in the absence of injury. We tested this by expressing in astrocytes a constitutively active Orai mutant that has a glycine to methionine substitution (Orai^G170M^) in the channel hinge that forces the channel into an open conformation.^43^ Transcriptional reporter of intracellular Ca^2+^-mediated GFP and GFP/RFP ratio was significantly higher in Orai^G170M^-expressing astrocytes compared with nonexpressing controls (Fig. 5A,B), demonstrating that Orai^G170M^ increases astrocyte Ca^2+^ signaling without injury. Strikingly, animals with astrocyte-specific Orai^G170M^ expression exhibited thermal allodynia even when uninjured, as their number of jumps at 38°C was similar to 7-day injured controls and significantly greater than uninjured controls (Fig. 5C). This significant increase in jumping was not seen in uninjured animals expressing a wildtype *Orai* transgene (*Orai*^*+*^), suggesting astrocyte Ca^2+^ influx is specifically responsible for the hypersensitivity. Collectively, our results demonstrate that astrocyte SOCE is both necessary and sufficient for development of thermal allodynia in adult *Drosophila*, and that astrocyte SOCE-mediated Ca^2+^ signaling is an essential component of the transition from acute injury to chronic pain.

## 4. Discussion

Recent findings demonstrate that Ca^2+^ influx through Orai1 SOCE channels in mouse astrocytes drives gliotransmitter release and tonic inhibition of pyramidal CA1 neurons, defining a role for astrocyte SOCE in physiological neural circuitry and function.^40^ Astrocyte Orai1 channels also drive neuroinflammatory responses in a mouse model of inflammatory depressive behaviors.^26^ Our results now demonstrate for the first time that astrocyte SOCE is also activated in response to nerve injury and is essential for CS. Store-operated Ca2+ entry may, therefore, play fundamental and highly conserved roles in multiple aspects of astrocyte physiology.

It is notable that our TRIC Ca^2+^ assays revealed a relatively narrow period of enhanced astrocyte Ca^2+^ signaling 3 to 4 days postinjury. This is consistent with data from mice showing that partial sciatic nerve ligation results in an increased cortical astrocyte Ca^2+^ transients between 3 and 6 days postinjury, with a return to basal frequency after day 6.^18^ The timing of allodynia development is also similar between *Drosophila* and mouse, with maximal allodynia 6 to 9 days postinjury and persistence for several weeks.^18,19^ Thus, enhanced astrocyte Ca^2+^ signaling may be an early and temporally confined response during the progression from acute injury to CS. This may have important therapeutic implications, because inhibiting astrocyte Ca^2+^ signals or downstream targets soon after nerve injury may effectively prevent development of chronic pain. We also found that TRIC-reported astrocyte Ca^2+^ responses were not confined to the second VNC bulbs where sensory nerves from the injured leg innervate, but instead were distributed throughout the VNC. Astrocytes form interconnected networks throughout the CNS via gap junctions that propagate signals including Ca^2+^,^29,31^ and connexin-43 proteins that can form gap junctions are required for neuropathic pain after acute injury in mice.^9^ Consistent with this, we found that astrocyte-specific suppression of the *Drosophila* gap junction component innexin-2 partially suppressed thermal allodynia following (Supplemental Figure S4, http://links.lww.com/PR9/A429).

Release of ER Ca^2+^ by IP-_3_Rs in astrocytes is clearly important for CS based on our results and prior findings in mice.^18^ IP_3_R-mediated Ca^2+^ release results in “somatic” astrocyte Ca^2+^ signals that occur throughout the cell body.^4^ It was previously thought that these IP_3_R-mediated Ca^2+^ responses downstream of metabotropic receptor activation constitute the primary mechanism by which astrocytes respond to synaptic activity. However, recent evidence suggests that “microdomain” Ca^2+^ signals confined to thin astrocytic processes are also essential components of Ca^2+^-dependent astrocyte information processing.^2,5,28,34,39^ Our major finding that astrocyte SOCE is essential for CS is highly significant in this regard, because SOCE produces microdomain Ca^2+^ signals that can activate downstream signaling processes independently of Ca^2+^ that is released by IP_3_Rs.^10,30^ It is likely that SOCE in astrocytes is activated subsequent to IP_3_R-mediated ER Ca^2+^ release, and the combination of IP_3_R-mediated somatic Ca^2+^ signals and SOCE-mediated microdomain Ca^2+^ signals may provide a robust, multifaceted Ca^2+^ signaling paradigm that results in highly efficient and specific activation of astrocyte functions to drive CS. However, a limitation of the TRIC reporter is that it cannot report precise spatiotemporal dynamics of intracellular Ca^2+^ signals. Live-imaging of astrocyte Ca^2+^ signals in the adult *Drosophila* CNS will be required to fully test this possibility, but our attempts at this have proven extremely challenging and thus-far unsuccessful.

Our findings demonstrate that astrocyte SOCE is required not only for the development of thermal allodynia but also for the neural circuitry changes that occur postinjury as revealed by VNC GABA immunoreactivity. Loss of GABAergic neurons after injury likely removes tonic inhibition of nociceptive circuits, resulting in hyperexcitability associated with allodynia.^3^ Loss of GABAergic neurons postinjury in flies is due to apoptosis,^17^ although it is not clear how apoptosis of these neurons is activated. We cannot determine from our experiments whether SOCE-regulated astrocyte function is directly responsible for GABAergic neuron loss, or whether astrocyte function is part of a cascade of events that results in neuronal apoptosis. A major goal moving forward is to define astrocyte SOCE targets, and how these targets regulate astrocyte functions that are essential for CS and neuropathic pain. Store-operated Ca2+ entry in cortical mouse astrocytes drives exocytic release of the gliotransmitters adenosine triphosphate and D-serine,^40^ and a similar function of SOCE activated by nerve injury may alter synaptic function within nociceptive circuits. Astrocytes also secrete inflammatory cytokines in response to Ca^2+^ signals,^14,23^ and cytokines may also modulate neuronal function or could induce GABAergic neuron apoptosis. Interestingly, it was recently shown in mice that nerve injury–induced SOCE in microglia is required for neuropathic pain due to SOCE-dependent microglial cytokine production.^41^ Although flies do not have the equivalent of microglia, SOCE-mediated cytokine release may be a major glial cell function in the pathogenesis of neuropathic pain.

A strength of our experiments is that suppression of Orai channels as well as Stim proteins that activate these channels yielded similar phenotypes with respect to suppression of injury-induced astrocyte Ca^2+^ signaling, thermal allodynia, and GABAergic neuron loss. *Drosophila* loss-of-function *Stim* and *Orai* mutants are available but are developmentally lethal, precluding testing these mutants for injury-induced phenotypes in adult animals. We instead used *in vivo* RNAi because it allows for targeted, astrocyte-specific suppression, and all the RNAi constructs used in our experiments were previously validated for specific and effective target suppression.^17,32,36^ And although RNAi rescue experiments would demonstrate specificity, these experiments are not feasible with the RNAi constructs used.^13^ An unexpected result in our experiments was that astrocyte Orai suppression in uninjured animals resulted in marginally increased astrocyte Ca^2+^ signaling, increased jumping at 38°C, and decreased GABA immunoreactivity. One possibility is that additional Ca^2+^ signaling pathways or channels are functionally upregulated when Orai channels are suppressed. To this end, it was shown that Waterwitch (Wtrw) and TrpML Ca^2+^ channels mediate somatic and microdomain astrocyte Ca^2+^ signals in *Drosophila*, respectively,^24,25^ and we found that astrocyte-specific suppression of these channels also partially attenuated thermal allodynia in flies (Supplemental Figure S5, http://links.lww.com/PR9/A429). It will be interesting to determine in future experiments whether these channels function in coordination with or independently of SOCE during CS signaling. It is also possible that suppression of Orai channels in astrocytes throughout development, as expected with the *alarm-GAL4* driver used in our experiments,^11^ may affect astrocyte physiology independently of nerve injury. Although this would not preclude our findings that astrocyte SOCE is required for the transition from nerve injury to neuropathic pain, it is a possibility that we plan to pursue in future studies. We also observed moderately large effect sizes (0.15–0.20) in allodynia experiments (Figs. 3A and 5C), likely reflecting inherent variability in animal behavior.

In conclusion, our results demonstrate that astrocyte SOCE is an essential component of the neural adaptations that lead to CS and neuropathic pain after acute nerve injury. These findings may drive the development of new, nonopioid therapeutics for neuropathic pain. Our findings further demonstrate that SOCE is an essential and highly conserved component of astrocyte function in neural physiology and pathogenesis. This will contribute significantly to our understanding of the complexity and diversity of Ca^2+^ signaling processes in astrocytes.

## Disclosures

The authors have no conflict of interest to declare.

## Supplemental digital content

Supplemental digital content associated with this article can be found online at http://links.lww.com/PR9/A429.
